# A new generalization of Apostol type Hermite–Genocchi polynomials and its applications

**DOI:** 10.1186/s40064-016-2357-4

**Published:** 2016-06-24

**Authors:** Serkan Araci, Waseem A. Khan, Mehmet Acikgoz, Cenap Özel, Poom Kumam

**Affiliations:** Department of Economics, Faculty of Economics, Administrative and Social Sciences, Hasan Kalyoncu University, 27410 Gaziantep, Turkey; Department of Mathematics, Integral University, Lucknow, 226026 India; Department of Mathematics, Faculty of Arts and Science, University of Gaziantep, 27310 Gaziantep, Turkey; Department of Mathematics, Dokuz Eylül University, 35160 Buca\Izmir, Turkey; Department of Mathematics, Faculty of Sciences, University of King Abdulaziz, Jeddah, Saudi Arabia; Theoretical and Computational Science Center (TaCS) and Department of Mathematics, Faculty of Science, King Mongkut’s University of Technology Thonburi (KMUTT), 126 Pracha Uthit Road, Bang Mod, Thung Khru, Bangkok, 10140 Thailand; Department of Medical Research, China Medical University Hospital, China Medical University, Taichung, 40402 Taiwan

**Keywords:** Hermite polynomials, Genocchi polynomials, Hermite–Genocchi polynomials, Apostol-Genocchi polynomials, Summation formulae, Symmetric identities, 11B68, 05A10, 05A15

## Abstract

By using the modified Milne-Thomson’s polynomial given in Araci et al. (Appl Math Inf Sci 8(6):2803–2808, 2014), we introduce a new concept of the Apostol Hermite–Genocchi polynomials. We also perform a further investigation for aforementioned polynomial and derive some implicit summation formulae and general symmetric identities arising from different analytical means and generating functions method. The results obtained here are an extension of Hermite–Bernoulli polynomials (Pathan and Khan in Mediterr J Math 12:679–695, 2015a) and Hermite–Euler polynomials (Pathan and Khan in Mediterr J Math 2015b, doi:10.1007/s00009-015-0551-1) to Apostol type Hermite–Genocchi polynomials defined in this paper.

## Background

Recently, the generalizations of Apostol-Bernoulli, Apostol-Euler and Apostol-Genocchi polynomials have been studied and investigated in Milovanović and Rassias (2014), Borwein and Erdelyi (1995), Agarwal (2014), Choi and Agarwal (2014), Srivastava et al. (2014), Agarwal (2012), Luo et al. (2014), Agarwal and Koul (2003), Apostol (1951), Araci (2014), Araci et al. (2014a, b), Bell (1934), Dattoli et al. (1999), Dere and Simsek (2015), Dere et al. (2013), Guo and Qi (2002), Gaboury and Kurt (2012), He et al. (2015), Jolany et al. (2013), Khan et al. (2008), Khan (2015, 2016a, b), Kim and Hu (2012), Kim and Adiga (2004), Kim (2007, 1999), Kurt and Kurt (2011), Luo et al. (2003a, b), Luo (2006, 2009, 2011), Luo and Srivastava (2005, 2011, 2006), Milne Thomsons (1933), Pathan and Khan (2014a, b, 2015a, b, c, d), Srivastava and Manocha (1984), Srivastava (2000, 2011), Yang (2008), Zhang and Yang (2008). The generalized Apostol-Bernoulli polynomials $B_{n}^{(\alpha )}(x;\lambda )$ of order $\alpha \in {\mathbb {C}}$, the generalized Apostol-Euler polynomials $E_{n}^{(\alpha )}(x;\lambda )$ of order $\alpha \in {\mathbb {C}}$ and the generalized Apostol-Genocchi polynomials $G_{n}^{(\alpha )}(x;\lambda )$ of order $\alpha \in {\mathbb {C}}$ are defined, respectively, by the following generating functions:

1 $$\begin{aligned}&\left( \frac{t}{\lambda e^{t}-1}\right) ^{\alpha }e^{xt}=\sum \limits _{n=0}^{\infty }B_{n}^{(\alpha )}(x;\lambda )\frac{t^{n}}{ n!}, (|t+\log \lambda |<2\pi ,1^{\alpha }:=1) \end{aligned}$$

2 $$\begin{aligned}&\left( \frac{2}{\lambda e^{t}+1}\right) ^{\alpha }e^{xt}=\sum \limits _{n=0}^{\infty }E_{n}^{(\alpha )}(x;\lambda )\frac{t^{n}}{ n!}, (|t+\log \lambda |<\pi ,1^{\alpha }:=1) \end{aligned}$$

and

3 $$\left( \frac{2t}{\lambda e^{t}+1}\right) ^{\alpha }e^{xt}=\sum \limits _{n=0}^{\infty }G_{n}^{(\alpha )}(x;\lambda )\frac{t^{n}}{ n!}, (|t+\log \lambda |<\pi ,1^{\alpha }:=1)$$

where, if we take $x=0$ in the above, we have

$$B_{n}^{(\alpha )}(0;\lambda ):=B_{n}^{(\alpha )}(\lambda ), E_{n}^{(\alpha )}(0;\lambda ):=E_{n}^{(\alpha )}(\lambda ){\text { and }} G_{n}^{(\alpha )}(0;\lambda ):=G_{n}^{(\alpha )}(\lambda )$$

calling Apostol-Bernoulli numbers of order $\alpha$, Apostol-Euler numbers of order $\alpha$ and Apostol-Genocchi numbers of order $\alpha$, respectively. Also,

$$B_{n}^{(\alpha )}(x):=B_{n}^{(\alpha )}(x;1), E_{n}^{(\alpha )}(x):=E_{n}^{(\alpha )}(x;1){\text { and }} G_{n}^{(\alpha )}(x)=G_{n}^{(\alpha )}(x;1).$$

             

See Dere et al. (2013), He et al. (2015), Jolany et al. (2013), Luo (2009), Luo and Srivastava (2005), Luo and Srivastava (2011) and Luo and Srivastava (2006) for a systematic work about the Apostol type polynomials.

Dere and Simsek (2015) gave a new class of the Milne-Thomson’s polynomials $\Phi _{n}^{(\alpha )}(x)$ as $\Phi _{n}^{(\alpha )}(x,y)$ of degree *n* and order $\alpha$ by means of the following generating function:

4 $$\sum \limits _{n=0}^{\infty }\Phi _{n}^{(\alpha )}(x,y)\frac{t^{n}}{n!} =f(t,\alpha )e^{xt+h(t,y)}$$

where $f(t,\alpha )$ is a function of *t* and integer $\alpha$. Observe that $\Phi _{n}^{(\alpha )}(x,0)=\Phi _{n}^{(\alpha )}(x)$ (see Luo and Srivastava 2006 for details). From here, setting $f(t,\alpha )=\left( \frac{2t}{\lambda e^{t}+1} \right) ^{\alpha }$ in (4) gives

5 $$\sum \limits _{n=0}^{\infty }G_{n}^{(\alpha )}(x,y;\lambda )\frac{t^{n}}{n!} =\left( \frac{2t}{\lambda e^{t}+1}\right) ^{\alpha }e^{xt+h(t,y)}$$

where $G_{n}^{(\alpha )}(x,y;\lambda )$ denotes the Apostol-Genocchi polynomials of higher order $\alpha$ based on Milne-Thomson’s polynomials.

It immediately follows from (4) and (5) that

$$G_{n}^{(\alpha )}(0,0;\lambda ) := G_{n}^{(\alpha )}(\lambda ).$$

Taking $h\left( t,y\right) =yt^{2}$ in (5) gives

6 $$\sum \limits _{n=0}^{\infty }{}_{H}G_{n}^{(\alpha )}(x,y;\lambda )\frac{t^{n}}{ n!}=\left( \frac{2t}{\lambda e^{t}+1}\right) ^{\alpha }e^{xt+yt^{2}}$$

where $_{H}G_{n}^{(\alpha )}(x,y;\lambda )$ is called generalized Apostol-Hermite Genocchi polynomials (see Gaboury and Kurt 2012). In the case $\alpha =1$ in (6), it reduces to Apostol-Hermite Genocchi polynomials defined by Dattoli et al. (1999) in the following form:

7 $$\sum \limits _{n=0}^{\infty }{}_{H}G_{n}(x,y)\frac{t^{n}}{n!}=\frac{2t}{e^{t}+1 }e^{xt+yt^{2}}.$$

Dattoli et al. (1999) and Luo et al. (2003a, b) gave the generalization of Bernoulli and Euler polynomials with *a* and *b* parameters, as follows:

8 $$\begin{aligned}&\sum \limits _{n=0}^{\infty }B_{n}(a,b)\frac{t^{n}}{n!}=\frac{t}{b^{t}-a^{t}},\quad \left( \left| t\log \frac{b}{a}\right| <2\pi \right) \end{aligned}$$

9 $$\begin{aligned}&\sum \limits _{n=0}^{\infty }E_{n}(a,b)\frac{t^{n}}{n!}=\frac{2}{b^{t}+a^{t}},\quad \left( \left| t\log \frac{b}{a}\right| <\pi \right) . \end{aligned}$$

Let *a* and *b* be positive integers. The generalized Apostol-Genocchi polynomials with the parameters *a*, *b* and *c* are given by means of the following generating function, i.e., a Taylor expansion about $t=0$:

10 $$\sum \limits _{n=0}^{\infty }G_{n}(x;a,b,c;\lambda )\frac{t^{n}}{n!}=\frac{2t}{ \lambda b^{t}+a^{t}}c^{xt}\quad ({\text {see}}\, {\text {Jolany et al. 2013}}).$$

For a real or complex parameter $\alpha$, the Apostol-Genocchi polynomials $G_{n}^{\left( \alpha \right) }(x;a,b,c;\lambda )$ of order $\alpha$ with parameters *a*, *b* and *c* are defined by means of the following generating function:

$$\sum \limits _{n=0}^{\infty }G_{n}^{\left( \alpha \right) }(x;a,b,c;\lambda ) \frac{t^{n}}{n!}=\left( \frac{2t}{\lambda b^{t}+a^{t}}\right) ^{\alpha }c^{xt}$$

from which it follows that $G_{n}^{\left( 1\right) }(x;a,b,c;\lambda ):=G_{n}(x;a,b,c;\lambda )$ cf. Jolany et al. (2013).

### **Definition 1**

Let *c* be positive integer. The generalized 2-variable 1-parameter Hermite Kamp’e de Feriet polynomials $H_{n}(x,y,c)$ for nonnegative integer *n* are stated by

11 $$\sum \limits _{n=0}^{\infty }H_{n}(x,y,c)\frac{t^{n}}{n!}=c^{xt+yt^{2}}$$

which is an extention of 2-variable Hermite Kamp’e de Feriet polynomials $H_{n}(x,y)$ defined by

12 $$\sum \limits _{n=0}^{\infty }H_{n}(x,y)\frac{t^{n}}{n!}=e^{xt+yt^{2}} \quad {\text{ (see Bell 1934; Pathan and Khan 2015a, b).}}$$

It immediately follows from Definition 1 that

$$H_{n}(x,y,e):=H_{n}(x,y).$$

and by (11), we have

13 $$H_{n}(x,y,c)=\sum \limits _{j=0}^{[\frac{n}{2}]}\left( {\begin{array}{c}n\\ j\end{array}}\right) (\log c)^{n-j}x^{n-2j}y^{j}\quad {\text { (see Pathan and Khan 2015a, b).}}$$

Motivated by their importance and potential for applications in certain problems in number theory, combinatorics, classical and numerical analysis and other fields of applied mathematics, several kinds of some special numbers and polynomials were recently studied by many authors (see Milovanović and Rassias 2014; Borwein and Erdelyi 1995; Agarwal 2014; Choi and Agarwal 2014; Srivastava et al. 2014; Agarwal 2012; Luo et al. 2014; Agarwal and Koul 2003; Apostol 1951; Araci 2014; Araci et al. 2014a, b; Bell 1934; Dattoli et al. 1999; Dere and Simsek 2015; Dere et al. 2013; Guo and Qi 2002; Gaboury and Kurt 2012; He et al. 2015; Jolany et al. 2013; Khan et al. 2008; Khan 2015, 2016a, b; Kim and Hu 2012; Kim and Adiga 2004; Kim 2007, 1999; Kurt and Kurt 2011; Luo et al. 2003a, b; Luo 2006, 2009, 2011; Luo and Srivastava 2005, 2011, 2006; Milne Thomsons 1933; Pathan and Khan 2014a, b, 2015a, b, c, d; Srivastava and Manocha 1984; Srivastava 2000, 2011; Yang 2008; Zhang and Yang 2008).

In Kurt and Kurt (2011), Kurt and Kurt first introduced the definition of Hermite–Apostol-Genocchi polynomials and derived some explicit formulas. Gaboury and Kurt (2012) also gave the generating function of Hermite–Apostol-Genocchi polynomials with three parameters. Their definitions are motivated us to write this paper. In summary, we introduce a new family of the generalized Apostol type Genocchi polynomials $G_{n}^{(\alpha )}(x,y;a,b,c;\lambda )$ as Definition 2 in the next section, which generalizes the concepts stated above and then research their basic properties and relationships with Genocchi numbers $G_{n}$, Genocchi polynomials $G_{n}(x)$ and the generalized Apostol Genocchi numbers $G_{n}(a,b;\lambda )$, generalized Apsotol Genocchi polynomials $G_{n}(x;a,b,c;\lambda )$ of Jolany et al. (2013), Hermite–Genocchi polynomial ${}_{H}G_{n}(x,y)$ of Dattoli et al. (1999) and generalized Apostol Hermite–Genocchi polynomials ${}_{H}G_{n}^{(\alpha )}(x,y;\lambda ).$ The remainder of this paper is organized as follows: We modify generating functions for the Milne-Thomson’s polynomials as defined in Luo and Srivastava (2006) and derive some identities related to Hermite polynomials and Genocchi polynomials. Some implicit summation formulae and general symmetric identities are derived arising from different analytical means and applying generating functions. These results extend some known summations and identities of Hermite–Bernoulli, Euler and Hermite–Genocchi polynomials studied earlier by Dattoli et al. (1999), Jolany et al. (2013), Khan (2015, 2016a, b), Luo (2009, 2011), Pathan and Khan (2014a, 2015a), Yang (2008), Zhang and Yang (2008).

## On the generalized Apostol type Hermite–Genocchi polynomials

In this section, by (4) and $f(t,\alpha ;\lambda )=\left( \frac{2t}{\lambda b^{t}+a^{t}}\right) ^{\alpha }$, we derive a new class of Apostol Hermite–Genocchi polynomials and investigate its properties. Now we start at the following definition.

### **Definition 2**

Let *a*, *b* and *c* be positive integers with the condition $a\ne b$. A new generalization of Apostol-Genocchi polynomials $G_{n}^{(\alpha )}(x,\nu ;a,b,c;\lambda )$ for nonnegative integer *n* is defined by

14 $$\begin{aligned}&\sum \limits _{n=0}^{\infty }G_{n}^{(\alpha )}(x,y;a,b,c;\lambda )\frac{t^{n}}{ n!}=\left( \frac{2t}{\lambda b^{t}+a^{t}}\right) ^{\alpha }c^{xt+h(t,y)} \\&\quad \left( |t|<\left| \frac{\log (-\lambda )}{\log (\frac{b}{a})}\right|; a\in {\mathbb {C}} \setminus \{0\},b,c\in {\mathbb {R}} ^{+}; 1^{\alpha }:=1\right) . \end{aligned}$$

Setting $h(t,y)=yt^{2}$ in (14), we get the following corollary.

### **Corollary 1**

*Leta*, *bandcbe positive integers with the condition*$a\ne b$. *The generalized Apostol Hermite–Genocchi polynomials*${}_{H}G_{n}^{(\alpha )}(x,y;a,b,c;\lambda )$*for nonnegative integernare defined by* Gaboury and Kurt (2012)

15 $$\begin{aligned}&\sum \limits _{n=0}^{\infty }{}_{H}G_{n}^{(\alpha )}(x,y;a,b,c;\lambda )\frac{ t^{n}}{n!}=\left( \frac{2t}{\lambda b^{t}+a^{t}}\right) ^{\alpha }c^{xt+yt^{2}} \\&\quad \left( \left| t\right| <\left| \frac{\log (-\lambda )}{\log ( \frac{b}{a})}\right| ; a\in {\mathbb {C}} \setminus \{0\},b,c\in {\mathbb {R}} ^{+};1^{\alpha }:=1\right) . \end{aligned}$$

For $\alpha =1$ in (15), we have

16 $$\sum \limits _{n=0}^{\infty }{_{H}G_{n}}(x,y;a,b,c;\lambda )\frac{t^{n}}{n!}= \frac{2t}{\lambda b^{t}+b^{t}}c^{xt+yt^{2}} \quad { ({\text {see}}\,{\text {Gaboury}}\,{\text {and}}\,{\text {Kurt}}\,2012).}$$

In the case $x=0$ in (15), we see that

17 $$_{H}G_{n}^{(\alpha )}(0,y;a,b,c;\lambda )=\sum \limits _{k=0}^{[\frac{n}{2}]} \frac{n!}{k!(n-2k)!}(\log c)^{k}G_{n-2k}^{(\alpha )}(a,b;\lambda )y^{k}.$$

Also in the case $x=y=0$ and $c=1$ in Definition 1, it leads to the extension of the generalized Apostol-Genocchi numbers denoted by $G_{n}^{(\alpha )}(a,b;\lambda )$ for nonnegative integer *n* defined earlier in Jolany et al. (2013) and

18 $$G_{n}^{(\alpha +\beta )}(a,b;\lambda )=\sum _{k=0}^{n}\left( {\begin{array}{c}n\\ k\end{array}}\right) G_{k}^{(\alpha )}(a,b;\lambda )G_{n-k}^{(\alpha )}(a,b;\lambda )$$

holds.

### **Corollary 2**

*Taking*$c=e$*in Eq.* (15), *we have* Gaboury and Kurt (2012)

19 $$\begin{aligned}&\sum \limits _{n=0}^{\infty }{}_{H}G_{n}^{(\alpha )}(x,y;a,b,e;\lambda )\frac{ t^{n}}{n!}=\left( \frac{2t}{\lambda b^{t}+a^{t}}\right) ^{\alpha }e^{xt+yt^{2}} \\&\quad \left( \left| t\right| <\left| \frac{\log (-\lambda )}{\log ( \frac{b}{a})}\right| ; a\in {\mathbb {C}} \setminus \{0\},b\in {\mathbb {R}}^{+};1^{\alpha }:=1\right) . \end{aligned}$$

By using Corollary 1, we state the following theorem.

### **Theorem 1**

*Leta*, *bandcbe positive integers with the rule*$a\ne b$. *For*$x\in {\mathbb {R}}$*and*$n\ge 0$. *Then we have*

20 $$\begin{aligned}&{}_{H}G_{n}^{(\alpha )}(x,y;1,e,e;\lambda ) :={}_{H}G_{n}^{(\alpha )}(x,y;\lambda ), {}_{H}G_{n}^{(\alpha )}(0,0;a,b,1;\lambda ) := G_{n}^{(\alpha )}(a,b;\lambda ), \\&{}_{H}G_{n}^{(\alpha )}(0,0;1,e,1,1) :=G_{n}^{(\alpha )}, {} _{H}G_{n}^{(1)}(0,0;a,b,1,1) := G_{n}(a,b) \end{aligned}$$

21 $$\begin{aligned}&{}_{H}G_{n}^{(\alpha +\beta )}(x+y,z+u;a,b,c;\lambda ) =\sum \limits _{k=0}^{n}\left( {\begin{array}{c}n\\ k\end{array}}\right) {}_{H}G_{n-k}^{(\alpha )}(y,z;a,b,c;\lambda ){}_{H}G_{k}^{(\beta )}(x,u;a,b,c;\lambda ) \end{aligned}$$

22 $$\begin{aligned}&{}_{H}G_{n}^{(\alpha )}(x+z,y;a,b,c;\lambda ) =\sum \limits _{k=0}^{n}\left( {\begin{array}{c}n\\ k\end{array}}\right) G_{n-k}^{(\alpha )}(z;a,b,c;\lambda )H_{k}(x,y;c). \end{aligned}$$

### Proof

The expressions stated in (20) are obvious from their generating functions. By using Definition 2, we have

$$\begin{aligned}&\sum \limits _{n=0}^{\infty }{}_{H}G_{n}^{(\alpha +\beta )}(x+y,z+u;a,b,c;\lambda )\frac{t^{n}}{n!} \\&\quad =\left( \sum \limits _{n=0}^{\infty }{}_{H}G_{n}^{(\alpha )}(y,z;a,b,c;\lambda )\frac{t^{n}}{n!}\right) \left( \sum \limits _{n=0}^{\infty }{}_{H}G_{n}^{(\beta )}(x,u;a,b,c;\lambda )\frac{ t^{n}}{n!}\right) \\&\quad =\sum \limits _{n=0}^{\infty }\left( \sum \limits _{k=0}^{n}{}\left( {\begin{array}{c}n\\ k\end{array}}\right) {} _{H}G_{k}^{(\beta )}(x,u;a,b,c;\lambda )_{H}G_{n-k}^{(\alpha )}(y,z;a,b,c;\lambda )\right) \frac{t^{n}}{n!}. \end{aligned}$$

B comparing the coefficients of $\frac{t^{n}}{n!}$, we get the Eq. (21). By the similar way, we readily derive the Eq. (22). Hence, we complete the proof of theorem.□

## Implicit summation formulae on the generalized Apostol type Hermite–Genocchi polynomials

We give here implicit summation formulae for Apostol Hermite–Genocchi polynomials. We now begin with the following theorem.

### **Theorem 2**

*Leta*, *bandcpositive integers, by*$a\ne b$. *Then, for*$x,y\in{\mathbb{R}}$*and*$m,n\ge 0$, *we have*

23 $$\begin{aligned} {}_{H}G_{m+n}^{(\alpha )}(z,y;a,b,c;\lambda )=\sum _{s=0}^{m}\sum \limits _{k=0}^{n}\left( {\begin{array}{c}m\\ s\end{array}}\right) \left( {\begin{array}{c}n\\ k\end{array}}\right) \left( \log c\right) ^{s+k}(z-x)^{s+k}{}_{H}G_{m+n-s-k}^{(\alpha )}(x,y;a,b,c;\lambda ) . \end{aligned}$$

### Proof

We first replace *t* by $t+u$ and rewrite the generating function (15) as

24 $$\begin{aligned}&\left( \frac{2(t+u)}{\lambda b^{t+u}+a^{t+u}}\right) ^{\alpha }c^{y(t+u)^{2}} \\&\quad =c^{-x(t+u)}\sum _{n=0}^{\infty }\sum \limits _{m=0}^{\infty }{}_{H}G_{m+n}^{(\alpha )}(x,y;a,b,c;\lambda )\frac{t^{n}}{n!}\frac{u^{m}}{m! }. \end{aligned}$$

Replacing *x* by *z* in (24), we have

25 $$\begin{aligned}&c^{(z-x)(t+u)}\sum _{n=0}^{\infty }\sum \limits _{m=0}^{\infty }{}_{H}G_{n+m}^{(\alpha )}(x,y;a,b,c;\lambda )\frac{t^{n}}{n!}\frac{u^{m}}{m! } \\&\quad =\sum _{n=0}^{\infty }\sum \limits _{m=0}^{\infty }{}_{H}G_{n+m}^{(\alpha )}(z,y;a,b,c;\lambda )\frac{t^{n}}{n!}\frac{u^{m}}{m!}. \end{aligned}$$

By applying

$$\sum \limits _{N=0}^{\infty }f(N)\frac{(x+y)^{N}}{N!}=\sum _{n=0}^{\infty }\sum \limits _{m=0}^{\infty }f(n+m)\frac{x^{n}}{n!}\frac{y^{m}}{m!}\qquad [{\text {see}}\,{\text {Pathan and Khan}}\,(2015{\text {d}}), {\text {p.}} 52(2)]$$

to $c^{(z-x)(t+u)}$ in (25), we get

26 $$\begin{aligned}&\sum \limits _{N=0}^{\infty }\frac{[(z-x)(t+u)]^{N}}{N!}\sum _{n=0}^{\infty }\sum \limits _{m=0}^{\infty }{}_{H}G_{n+m}^{(\alpha )}(x,y;a,b,c)\frac{t^{n}}{ n!}\frac{u^{m}}{m!} \\&\quad =\sum _{n=0}^{\infty }\sum \limits _{m=0}^{\infty }{}_{H}G_{n+m}^{(\alpha )}(z,y;a,b,c;\lambda )\frac{t^{n}}{n!}\frac{u^{m}}{m!}. \end{aligned}$$

It follows from (26) that

27 $$\begin{aligned}&\sum _{k=0}^{\infty }\sum \limits _{s=0}^{\infty }\frac{\left( \log c\right) ^{s+k}(z-x)^{k+s}t^{k}u^{s}}{k!s!}\sum _{n=0}^{\infty }\sum \limits _{m=0}^{\infty }{}_{H}G_{m+n}^{(\alpha )}(x,y;a,b,c;\lambda ) \frac{t^{n}}{n!}\frac{u^{m}}{m!} \\&\quad =\sum _{n=0}^{\infty }\sum \limits _{m=0}^{\infty }{}_{H}G_{n+m}^{(\alpha )}(z,y;a,b,c;\lambda )\frac{t^{n}}{n!}\frac{u^{m}}{m!}. \end{aligned}$$

Replacing *n* by $n-k$ and *s* by $m-s$ and using the lemma in [44, p. 100 (1)] gives

28 $$\begin{aligned}&\sum _{n=0}^{\infty }\sum \limits _{m=0}^{\infty }\left( \sum _{k=0}^{\infty }\sum \limits _{s=0}^{\infty }\frac{\left( \log c\right) ^{s+k}(z-x)^{k+s}}{ k!s!}{}_{H}G_{m+n-k-s}^{(\alpha )}(x,y;a,b,c;\lambda )\right) \frac{t^{n}}{ (n-k)!}\frac{u^{m}}{(m-s)!} \\&\quad =\sum _{m=0}^{\infty }\sum \limits _{n=0}^{\infty }{}_{H}G_{n+m}^{(\alpha )}(z,y;a,b,c;\lambda )\frac{t^{n}}{n!}\frac{u^{m}}{m!}. \end{aligned}$$

By comparing the coefficients $t^{n}u^{m}$ in (28), we arrive at the desired result.□

### **Corollary 3**

*For*$m=0$*in* (23), *we have*

29 $${}_{H}G_{n}^{(\alpha )}(z,y;a,b,c;\lambda )=\sum \limits _{k=0}^{n}\left( {\begin{array}{c}n\\ k\end{array}}\right) \left( \log c\right) ^{k}(z-x)^{k}{}_{H}G_{n-k}^{(\alpha )}(x,y,a,b,c;\lambda ).$$

### **Corollary 4**

*Replacingzby*$z+x$*and taking*$y=0$*in* (23), *we get*

30 $$\begin{aligned} G_{m+n}^{(\alpha )}(z+x;a,b,c;\lambda )=\sum _{s=0}^{m}\sum \limits _{k=0}^{n} \left( {\begin{array}{c}m\\ s\end{array}}\right) \left( {\begin{array}{c}n\\ k\end{array}}\right) \left( \log c\right) ^{s+k}z^{k+s}G_{m+n-k-s}^{(\alpha )}(x;a,b,c;\lambda ). \end{aligned}$$

*Moreover, taking*$z=0$*in* (23), *we have*

31 $$\begin{aligned} G_{m+n}^{(\alpha )}(y;a,b,c;\lambda )=\sum _{s=0}^{m}\sum \limits _{k=0}^{n} \left( {\begin{array}{c}m\\ s\end{array}}\right) \left( {\begin{array}{c}n\\ k\end{array}}\right) \left( \log c\right) ^{s+k}(-x)^{k+s}{}_{H}G_{m+n-k-s}^{(\alpha )}(x,y;a,b,c;\lambda ). \end{aligned}$$

We also derive additional results arising from Eq. (23), as follows.

### **Corollary 5**

*For*$y=0$*in* (23), *we have*

32 $$\begin{aligned} G_{m+n}^{(\alpha )}(z;a,b,c;\lambda )=\sum _{s=0}^{m}\sum \limits _{k=0}^{n} \left( {\begin{array}{c}m\\ s\end{array}}\right) \left( {\begin{array}{c}n\\ k\end{array}}\right) \left( \log c\right) ^{s+k}(z-x)^{k+s}G_{m+n-k-s}^{(\alpha )}(x;a,b,c;\lambda ). \end{aligned}$$

### **Corollary 6**

*For*$\alpha =1$*in* (23), *we have*

33 $$\begin{aligned} {}_{H}G_{k+l}(z,y;a,b,c;\lambda )=\sum _{s=0}^{m}\sum \limits _{k=0}^{n}\left( {\begin{array}{c}m \\ s\end{array}}\right) \left( {\begin{array}{c}n\\ k\end{array}}\right) \left( \log c\right) ^{s+k}(z-x)^{k+s}{}_{H}G_{m+n-k-s}(x,y;a,b,c;\lambda ). \end{aligned}$$

*where*${}_{H}G_{m+n}(z,y;a,b,c;\lambda )$*denotes the generalized Apostoltype Hermite–Genocchi polynomials*.

### **Theorem 3**

*Leta*, *band*$c$*be positive integers, by*$a\ne b$. *Then, for*$x,y\in{\mathbb{R}}$*and*$n\ge 0$, *we have*

34 $$\begin{aligned} {}_{H}G_{n}^{(\alpha )}(x+\alpha ,y;a,b,c;\lambda )=\sum \limits _{k=0}^{[ \frac{n}{2}]}\left( {\begin{array}{c}n\\ 2k\end{array}}\right) y^{k}(\log c)^{k}G_{n-2k}^{(\alpha )}(x;\frac{a}{c} ,\frac{b}{c},c;\lambda ) \end{aligned}$$

*where [.] is Gauss’ notation, and represents the maximum integer which does not exceed a number in the square brackets*.

### Proof

By the exponential generating function of the polynomial $_{H}G_{n}^{(\alpha )}\left( x+\alpha ,y;a,b,c;\lambda \right)$, we have

$$\begin{aligned} {}\sum \limits _{n=0}^{\infty }{}_{H}G_{n}^{(\alpha )}(x+\alpha ,y;a,b,c;\lambda )\frac{t^{n}}{n!}& = \left( \frac{2t}{\lambda b^{t}+a^{t}} \right) ^{\alpha }c^{(x+\alpha )t+yt^{2}} \\& = \left( \frac{2t}{\lambda (\frac{b}{c}){^{t}}+(\frac{a}{c})^{t}}\right) ^{\alpha }c^{xt}c^{yt^{2}} \\& = \left( \sum \limits _{n=0}^{\infty }G_{n}^{(\alpha )}(x;\frac{a}{c},\frac{b }{c},c;\lambda )\frac{t^{n}}{n!}\right) \left( \sum \limits _{n=0}^{\infty }y^{n}(\log c)^{n}\frac{t^{2n}}{n!}\right) \\&\sum \limits _{n=0}^{\infty }\left( \sum \limits _{k=0}^{[\frac{n}{2}]}\left( {\begin{array}{c}n \\ 2k\end{array}}\right) y^{k}(\log c)^{k}G_{n-2k}^{(\alpha )}(x;\frac{a}{c},\frac{b}{c} ,c;\lambda )\right) \frac{t^{n}}{n!}. \end{aligned}$$

Thus we get the desired result.□

### **Corollary 7**

*Taking*$\alpha =1$*in* (34) *gives*

$$\begin{aligned} _{H}G_{n}(x+1,y;a,b,c;\lambda )=\sum \limits _{k=0}^{[\frac{n}{2}]}\left( {\begin{array}{c}n\\ 2k \end{array}}\right) y^{k}(\log c)^{k}G_{n-2k}(x;\frac{a}{c},\frac{b}{c},c;\lambda ) \end{aligned}$$

*where [.] is Gauss’ notation, and represents the maximum integer which does not exceed a number in the square brackets*.

### **Theorem 4**

*Leta*, *bandcbe positive integers, by*$a\ne b$. *Then, for*$x,y\in{\mathbb{R}}$*and*$n\ge 0$, *we have*

35 $$\begin{aligned} {}_{H}G_{n}^{(\alpha )}(x,y;a,b,c;\lambda )=\sum \limits _{k=0}^{n}\left( {\begin{array}{c}n\\ k\end{array}}\right) G_{n-k}^{(\alpha )}(a,b;\lambda )H_{k}(x,y,c). \end{aligned}$$

### Proof

By (11) and (15), we have

$$\begin{aligned} \left( \frac{2t}{\lambda b^{t}+a^{t}}\right) ^{\alpha }c^{xt+yt^{2}}& = \sum \limits _{n=0}^{\infty }{}_{H}G_{n}^{(\alpha )}(x,y;a,b,c;\lambda ) \frac{t^{n}}{n!} \\ & = \left( \sum \limits _{n=0}^{\infty }G_{n}^{(\alpha )}(a,b;\lambda )\frac{ t^{n}}{n!}\right) \left( \sum \limits _{n=0}^{\infty }H_{n}(x,y;c)\frac{t^{n}}{ n!}\right) \\ & = \sum \limits _{n=0}^{\infty }\left( \sum \limits _{k=0}^{n}\left( {\begin{array}{c}n\\ k\end{array}}\right) G_{n-k}^{(\alpha )}(a,b;\lambda )H_{k}(x,y,c)\right) \frac{t^{n}}{n!}. \end{aligned}$$

Thus we complete the proof of theorem.□

### **Corollary 8**

*Putting*$c = e$*in* (35) *yields to*

$$\begin{aligned} {}_{H}G_{n}^{(\alpha )}(x,y;a,b,e;\lambda )=\sum \limits _{k=0}^{n}\left( {\begin{array}{c}n\\ k\end{array}}\right) G_{n-k}^{\left( \alpha \right) }(a,b;\lambda )H_{k}(x,y). \end{aligned}$$

### **Theorem 5**

*Leta*, *bandcbe positive integers, by*$a\ne b$. *Then, for*$x,y\in{\mathbb{R}}$*and*$n\ge 0$, *we have*

36 $$\begin{aligned} {}\frac{_{H}G_{n}^{(\alpha )}(x,y;a,b,c;\lambda )}{n!}=\sum \limits _{j=0}^{[ \frac{n}{2}]}\left( \sum \limits _{k=0}^{n-2j}\frac{(\log c)^{n-k-j}x^{n-k-2j}y^{j}}{j!(n-2j-k)!}\right) \frac{G_{k}^{(\alpha )}(a,b;\lambda )}{k!} \end{aligned}$$

*where [.] is Gauss’ notation, and represents the maximum integer which does not exceed a number in the square brackets.*

### Proof

Since

$$\begin{aligned} \left( \frac{2t}{\lambda b^{t}+a^{t}}\right) ^{\alpha }c^{xt+yt^{2}}=\left( \sum \limits _{k=0}^{\infty }G_{k}^{(\alpha )}(a,b;\lambda )\frac{t^{k}}{k!} \right) \left( \sum \limits _{n=0}^{\infty }x^{n}(\log c)^{n}\frac{t^{n}}{n!} \right) \left( \sum \limits _{j=0}^{\infty }y^{j}(\log c)^{j}\frac{t^{2j}}{j!} \right) \end{aligned}$$

we have

$$\begin{aligned} =\sum \limits _{n=0}^{\infty }\left( \sum \limits _{k=0}^{n}\left( {\begin{array}{c}n\\ k\end{array}}\right) (\log c)^{n-k}G_{k}^{(\alpha )}(a,b;\lambda )x^{n-k}\right) \frac{t^{n}}{n!}\left( \sum \limits _{j=0}^{\infty }y^{j}(\log c)^{j}\frac{t^{2j}}{j!}\right) . \end{aligned}$$

Replacing *n* by $n-2j$ in the right hand side, we have

37 $$\begin{aligned} \sum \limits _{n=0}^{\infty }{}_{H}G_{n}^{(\alpha )}(x,y;a,b,c;\lambda )\frac{ t^{n}}{n!}=\sum \limits _{n=0}^{\infty }\left( \sum \limits _{j=0}^{[\frac{n}{2} ]}\sum \limits _{k=0}^{n-2j}\left( {\begin{array}{c}n-2j\\ k\end{array}}\right) (\log c)^{n-k-j}G_{k}^{(\alpha )}(a,b;\lambda )x^{n-k-2j}y^{j}\right) \frac{t^{n}}{(n-2j)!j!}. \end{aligned}$$

Hence, our assertion follows from (37).□

### **Corollary 9**

*For*$y=0$*in* (36), *we get*

$$\begin{aligned} G_{n}^{(\alpha )}(x;a,b,c;\lambda )=\sum \limits _{k=0}^{n}\left( {\begin{array}{c}n\\ k\end{array}}\right) (\log c)^{n-k}G_{k}^{(\alpha )}(a,b;\lambda )x^{n-k}. \end{aligned}$$

*Moreover, setting*$x=0$*reduces* (17).

### **Theorem 6**

*Leta*, *bandcbe positive integers, by*$a\ne b$. *Then, for*$x,y\in{\mathbb{R}}$*and*$n\ge 0$, *we have*

38 $$\begin{aligned} {}_{H}G_{n}^{(\alpha )}(x+1,y;a,b,c;\lambda )=\sum \limits _{j=0}^{[\frac{n}{2} ]}\sum \limits _{k=0}^{n-2j}\left( {\begin{array}{c}n-2j\\ k\end{array}}\right) (\log c)^{n-k-j}y^{j}G_{k}^{(\alpha )}(x;a,b,c;\lambda ) \end{aligned}$$

*where [.] is Gauss’ notation, and represents the maximum integer which does not exceed a number in the square brackets*.

### Proof

It follows from (15) that

$$\begin{aligned} \sum \limits _{n=0}^{\infty }{}_{H}G_{n}^{(\alpha )}(x+1,y;a,b,c;\lambda ) \frac{t^{n}}{n!} & = \left( \frac{2t}{\lambda b^{t}+a^{t}}\right) ^{\alpha }c^{(x+1)t+yt^{2}} \\ & = \left( \sum \limits _{k=0}^{\infty }G_{k}^{(\alpha )}(x;a,b,c;\lambda ) \frac{t^{k}}{k!}\right) \left( \sum \limits _{n=0}^{\infty }(\log c)^{n}\frac{ t^{n}}{n!}\right) \left( \sum \limits _{j=0}^{\infty }y^{j}(\log c)^{j}\frac{ t^{2j}}{j!}\right) \\ & = \sum \limits _{n=0}^{\infty }\sum \limits _{k=0}^{n}\left( {\begin{array}{c}n\\ k\end{array}}\right) (\log c)^{n-k}G_{k}^{(\alpha )}(x;a,b,c;\lambda )\frac{t^{n}}{n!}\left( \sum \limits _{j=0}^{\infty }y^{j}(\log c)^{j}\frac{t^{2j}}{j!}\right) \\ & = \sum \limits _{n=0}^{\infty }\sum \limits _{j=0}^{\infty }\sum \limits _{k=0}^{n}\left( {\begin{array}{c}n\\ k\end{array}}\right) (\log c)^{n-k+j}G_{k}^{(\alpha )}(x;a,b,c;\lambda )\frac{t^{n+2j}}{n!j!} \\ & = \sum \limits _{n=0}^{\infty }\sum \limits _{j=0}^{[\frac{n}{2} ]}\sum \limits _{k=0}^{n-2j}\left( {\begin{array}{c}n-2j\\ k\end{array}}\right) (\log c)^{n-k-j}y^{j}G_{k}^{(\alpha )}(x;a,b,c;\lambda )\frac{t^{n}}{(n-2j)!j!}. \end{aligned}$$

Hence, our assertion completes the proof of theorem.□

### **Theorem 7**

*Letaandbbe positive integers, by*$a\ne b$. *Then, for*$x,y\in{\mathbb{R}}$*and*$n\ge 0$, *we have*

$$\begin{aligned} {}_{H}G_{n}^{(\alpha +1)}(x,y;a,b,e;\lambda )=\sum \limits _{k=0}^{n}\left( {\begin{array}{c}n\\ k\end{array}}\right) G_{n-k}(a,b;\lambda ){}_{H}G_{m}^{(\alpha )}(x,y;a,b,e;\lambda ). \end{aligned}$$

### Proof

It is proved by using

$$\begin{aligned} \frac{2t}{\lambda b^{t}+a^{t}}\left( \frac{2t}{\lambda b^{t}+a^{t}}\right) ^{\alpha }e^{xt+yt^{2}}=\frac{2t}{\lambda b^{t}+a^{t}}\sum \limits _{n=0}^{ \infty }{}_{H}G_{n}^{(\alpha )}(x,y;a,b,e;\lambda )\frac{t^{n}}{n!} \end{aligned}$$

and Cauchy product formula.□

### **Theorem 8**

*For arbitrary real or complex parameter*$\alpha$, *the following implicit summation formula involving generalized Apostol type Hermite–Genoccchi polynomials*${}_{H}G_{n}^{(\alpha )}(x,y;a,b,c;\lambda )$*holds true:*

39 $$\begin{aligned} {}_{H}G_{n}^{(\alpha )}(x+1,y;a,b,c;\lambda )=\sum \limits _{k=0}^{n}\left( {\begin{array}{c}n\\ k\end{array}}\right) (\log c)^{n-k}{}_{H}G_{k}^{(\alpha )}(x,y;a,b,c;\lambda ). \end{aligned}$$

### Proof

By (15), we have

$$\begin{aligned}&\sum \limits _{n=0}^{\infty }{}_{H}G_{n}^{(\alpha )}(x+1,y;a,b,c;\lambda ) \frac{t^{n}}{n!}-\sum \limits _{n=0}^{\infty }{}_{H}G_{n}^{(\alpha )}(x,y;a,b,c;\lambda )\frac{t^{n}}{n!} \\&\quad =\left( \frac{2t}{\lambda b^{t}+a^{t}}\right) ^{\alpha }c^{xt+yt^{2}}(c^{t}-1) \\&\quad =\left( \sum \limits _{k=0}^{\infty }{}_{H}G_{k}^{(\alpha )}(x,y;a,b,c;\lambda )\frac{t^{k}}{k!}\right) \left( \sum \limits _{n=0}^{\infty }(\log c)^{n}\frac{t^{n}}{n!}\right) -\sum \limits _{n=0}^{\infty }{}_{H}G_{n}^{(\alpha )}(x,y;a,b,c;\lambda )\frac{ t^{n}}{n!} \\&\quad =\sum \limits _{n=0}^{\infty }\sum \limits _{k=0}^{n}\left( {\begin{array}{c}n\\ k\end{array}}\right) (\log c)^{n-k}{}_{H}G_{k}^{(\alpha )}(x,y;a,b,c;\lambda )\frac{t^{n}}{n!} -\sum \limits _{n=0}^{\infty }{}_{H}G_{n}^{(\alpha )}(x,y;a,b,c;\lambda )\frac{ t^{n}}{n!}. \end{aligned}$$

By equating the coefficients of the like powers of $t^{n}$, we arrive at the desired result.□

### **Theorem 9**

*For arbitrary real or complex parameter*$\alpha$, *the following implicit summation formula involving generalized Apostol type Hermite Genocchi polynomials*${}_{H}G_{n}^{(\alpha )}(x,y;a,b,c;\lambda )$*holds true:*

40 $$\begin{aligned} \sum \limits _{k=0}^{n}\left( {\begin{array}{c}n\\ k\end{array}}\right) (\log ab)^{k}\alpha ^{k}{}_{H}G_{n-k}^{(\alpha )}(-x,y;a,b,c;\lambda )=(-1)^{n}{}_{H}G_{n}^{(\alpha )}(x,y;a,b,c;\lambda ) \end{aligned}$$

*and*

41 $$\begin{aligned} {}_{H}G_{n}^{(\alpha )}(\alpha -x,y;\frac{c}{a},\frac{c}{b},c;\lambda )=(-1)^{n}{}_{H}G_{n}^{(\alpha )}(x,y;a,b,c;\lambda ). \end{aligned}$$

### Proof

By (15), we have

$$\begin{aligned} c^{yt^{2}}\left[ \left( \frac{2t}{\lambda b^{t}+a^{t}}\right) ^{\alpha }(c^{xt}-(ab)^{\alpha t}c^{-xt})\right] =\sum \limits _{n=0}^{\infty }[1-(-1)^{n}]{}_{H}G_{n}^{(\alpha )}(x,y;a,b,c;\lambda )\frac{t^{n}}{n!} \end{aligned}$$

which is equivalent to

$$\begin{aligned}&\sum \limits _{n=0}^{\infty }{}_{H}G_{n}^{(\alpha )}(x,y;a,b,c;\lambda ) \frac{t^{n}}{n!}-\left( \sum \limits _{m=0}^{\infty }\alpha ^{m}(\log ab)^{m} \frac{t^{m}}{m!}\right) \sum \limits _{n=0}^{\infty }{}_{H}G_{n}^{(\alpha )}(-x,y;a,b,c;\lambda )\frac{t^{n}}{n!} \\&\quad =\sum \limits _{n=0}^{\infty }{}_{H}G_{n}^{(\alpha )}(x,y;a,b,c;\lambda ) \frac{t^{n}}{n!}-\left( \sum \limits _{n=0}^{\infty }\sum \limits _{m=0}^{n}\alpha ^{m}(\log ab)^{m}\right) {}_{H}G_{n-m}^{(\alpha )}(-x,y;a,b,c;\lambda )\frac{t^{n}}{(n-m)!} \\&\quad =\sum \limits _{n=0}^{\infty }[1-(-1)^{n}]{}_{H}G_{n}^{(\alpha )}(x,y;a,b,c;\lambda )\frac{t^{n}}{n!}. \end{aligned}$$

By equating coefficients of like powers of $t^{n}$, we complete (40). In order to show the proof of (41), it is sufficient to see that

$$\begin{aligned} c^{yt^{2}}\left[ \left( \frac{2t}{\lambda b^{t}+a^{t}}\right) ^{\alpha }c^{xt}-\left( \frac{2t}{\lambda (\frac{c}{a})^{t}+(\frac{c}{b})^{t}}\right) ^{\alpha t}c^{(\alpha -x)t}\right] =\sum \limits _{n=0}^{\infty }[1-(-1)^{n}]{}_{H}G_{n}^{(\alpha )}(x,y;a,b,c;\lambda )\frac{t^{n}}{n!} \end{aligned}$$

and

$$\begin{aligned}&\sum \limits _{n=0}^{\infty }{}_{H}G_{n}^{(\alpha )}(x,y;a,b,c;\lambda ) \frac{t^{n}}{n!}-\sum \limits _{n=0}^{\infty }{}_{H}G_{n}^{(\alpha )}(\alpha -x,y;\frac{c}{a},\frac{c}{b},c;\lambda )\frac{t^{n}}{n!} \\&\quad =\sum \limits _{n=0}^{\infty }[1-(-1)^{n}]{}_{H}G_{n}^{(\alpha )}(x,y;a,b,c;\lambda )\frac{t^{n}}{n!}. \end{aligned}$$

□

### **Corollary 10**

*Setting*$b=c=e$*and*$\lambda =a=1$*in* (40), *we have*

$$\begin{aligned} \sum \limits _{k=0}^{n}\left( {\begin{array}{c}n\\ k\end{array}}\right) \alpha ^{k}{}_{H}G_{n-k}^{(\alpha )}(-x,y;\lambda )=(-1)^{n}{}_{H}G_{n}^{(\alpha )}(x,y;\lambda ). \end{aligned}$$

### **Corollary 11**

*For*$b=c=e$*and*$a=1$*in* (40), *we have*

$$\begin{aligned} {}_{H}G_{n}^{(\alpha )}(\alpha -x,y;\lambda )=(-1)^{n}{}_{H}G_{n}^{(\alpha )}(x,y;\lambda ) \end{aligned}$$

*which is known as symmetry property of the generalized Hermite–Apostol Genocchi polynomials.*

## General symmetry identities

In this section, we investigate and derive symmetric identities for the generalized Apostol type Hermite–Genocchi polynomials ${}_{H}G_{n}^{(\alpha )}(x,y;a,b,c;\lambda )$ and Apostol Genocchi numbers $G_{n}^{(\alpha )}(a,b;\lambda )$. It turns out that some well known identities of Khan et al. (2008), Khan (2015, a), Milne Thomsons (1933), Pathan and Khan (2014a, b, 2015a, b, c), Srivastava (2011) and Yang (2008). As it has been mentioned in previous sections, $\alpha$ will be considered as an arbitrary real or a complex parameter.

### **Theorem 10**

*Leta*, *bandcbe positive integers, by*$a\ne b$. *Then, for*$x,y\in{\mathbb{R}}$*and*$n\ge 0$, *we have*

$$\begin{aligned}&\sum \limits _{k=0}^{n}\left( {\begin{array}{c}n\\ k\end{array}}\right) a^{n-k}b^{k}{}_{H}G_{n-k}^{(\alpha )}(bx,b^{2}y;A,B,c;\lambda ){}_{H}G_{k}^{(\alpha )}(ax,a^{2}y;A,B,c;\lambda ) \\ & = \sum \limits _{k=0}^{n}\left( {\begin{array}{c}n\\ k\end{array}}\right) b^{n-k}a^{k}{}_{H}G_{n-k}^{(\alpha )}(ax,a^{2}y;A,B,c;\lambda ){}_{H}G_{k}^{(\alpha )}(bx,b^{2}y;A,B,c;\lambda ). \end{aligned}$$

### Proof

Let us consider

42 $$\begin{aligned} g(t)=\left( \frac{(2t)^{2}}{(\lambda A^{at}+B^{at})(\lambda A^{bt}+B^{bt})} \right) ^{\alpha }c^{abxt+a^{2}b^{2}yt^{2}}. \end{aligned}$$

Then we see that *g*(*t*) is symmetric in *a* and *b*, and therefore we consider *g*(*t*) in two ways: Firstly

43 $$\begin{aligned} g(t) & = \sum \limits _{n=0}^{\infty }{}_{H}G_{n}^{(\alpha )}(bx,b^{2}y;A,B,c;\lambda )\frac{(at)^{n}}{n!}\sum \limits _{k=0}^{\infty }{}_{H}G_{k}^{(\alpha )}(ax,a^{2}y;A,B,c;\lambda )\frac{(bt)^{k}}{k!} \\ & = \frac{1}{\left( ab\right) ^{\alpha }}\sum \limits _{n=0}^{\infty }\sum \limits _{k=0}^{n}{}_{H}G_{n-k}^{(\alpha )}(bx,b^{2}y;A,B,c;\lambda ) \frac{a^{n-k}}{(n-k)!}{}_{H}G_{k}^{(\alpha )}(bx,b^{2}y;A,B,c;\lambda )\frac{ b^{k}t^{n}}{k!}. \end{aligned}$$

Secondly

$$\begin{aligned} g(t) & = \sum \limits _{n=0}^{\infty }{}_{H}G_{n}^{(\alpha )}(ax,a^{2}x;A,B,c;\lambda )\frac{(bt)^{n}}{n!}\sum \limits _{k=0}^{\infty }{}_{H}G_{k}^{(\alpha )}(bx,b^{2}y;A,B,c;\lambda )\frac{(at)^{k}}{k!}\\ & = \frac{1}{\left( ab\right) ^{\alpha }}\sum \limits _{n=0}^{\infty }\sum \limits _{k=0}^{n}{}_{H}G_{n-k}^{(\alpha )}(ax,a^{2}y;A,B,c;\lambda ) \frac{b^{n-k}}{(n-k)!}{}_{H}G_{k}^{(\alpha )}(bx,b^{2}y;A,B,c;\lambda )\frac{ a^{k}t^{n}}{k!}. \end{aligned}$$

By comparing the coefficients of $t^{n}$ on the right hand sides of two ways, we arrive at the desired result.□

### **Corollary 12**

*Setting*$b=1$*in Theorem*10*gives*

$$\begin{aligned}&\sum \limits _{k=0}^{n}\left( {\begin{array}{c}n\\ k\end{array}}\right) a^{n-k}{}_{H}G_{n-k}^{(\alpha )}(x,y;A,B,c;\lambda ){}_{H}G_{k}^{(\alpha )}(ax,a^{2}y;A,B,c;\lambda )\\&\qquad =\sum \limits _{k=0}^{n}\left( {\begin{array}{c}n\\ k\end{array}}\right) a^{k}{}_{H}G_{n-k}^{(\alpha )}(ax,a^{2}y;A,B,c;\lambda ){}_{H}G_{k}^{(\alpha )}(x,y;A,B,c;\lambda ) \end{aligned}$$

### **Theorem 11**

*Leta*, *bandcbe positive integeres, by*$a\ne b$. *Then, for*$x,y\in{\mathbb{R}}$*and*$n\ge 0$, *the following identity holds true:*

$$\begin{aligned}&\sum \limits _{k=0}^{n}\left( {\begin{array}{c}n\\ k\end{array}}\right) a^{n-k}b^{k}\sum \limits _{i=0}^{a-1}\sum \limits _{j=0}^{b-1}(-\lambda )^{i+j}{}_{H}G_{n-k}^{(\alpha )}\left( bx+\frac{ b}{a}i+j,b^{2}z;c;\lambda \right) G_{k}^{(\alpha )}(ay;c,\lambda )\\&\qquad =\sum \limits _{k=0}^{n}\left( {\begin{array}{c}n\\ k\end{array}}\right) b^{n-k}a^{k}\sum \limits _{i=0}^{b-1}\sum \limits _{j=0}^{a-1}(-\lambda )^{i+j}{}_{H}G_{n-k}^{(\alpha )}\left( ax+\frac{ a}{b}i+j,a^{2}z;c,\lambda \right) G_{k}^{(\alpha )}(by;c,\lambda ). \end{aligned}$$

### Proof

Let us first consider the following function:

$$\begin{aligned} g(t)=\frac{(2at)^{\alpha }(2bt)^{\alpha }(\lambda c^{abt}+1)^{2}c^{ab(x+y)t+a^{2}b^{2}zt^{2}}}{(\lambda c^{at}+1)^{\alpha +1}(\lambda c^{bt}+1)^{\alpha +1}} \end{aligned}$$

which equals to

$$\begin{aligned} g(t)=\left( \frac{2at}{\lambda c^{at}+1}\right) ^{\alpha }c^{abxt+a^{2}b^{2}zt^{2}}\left( \frac{\lambda c^{abt}+1}{\lambda c^{bt}+1} \right) \left( \frac{2bt}{\lambda c^{bt}+1}\right) ^{\alpha }c^{abyt}\left( \frac{\lambda c^{abt}+1}{\lambda c^{at}+1}\right) . \end{aligned}$$

From here, we have

$$\begin{aligned} & = \sum \limits _{n=0}^{\infty }\left( \sum \limits _{k=0}^{n}\left( {\begin{array}{c}n\\ k\end{array}}\right) a^{n-k}b^{k}\sum \limits _{i=0}^{a-1}\sum \limits _{j=0}^{b-1}(-\lambda )^{i+j}{}_{H}G_{n-k}^{(\alpha )}\left( ax+\frac{b}{a}i+j,b^{2}z;c,\lambda \right) G_{k}^{(\alpha )}(ay;c,\lambda )\right) \frac{t^{n}}{n!} \\&\sum \limits _{n=0}^{\infty }\left( \sum \limits _{k=0}^{n}\left( {\begin{array}{c}n\\ k\end{array}}\right) b^{n-k}a^{k}\sum \limits _{i=0}^{b-1}\sum \limits _{j=0}^{a-1}(-\lambda )^{i+j}{}_{H}G_{n-k}^{(\alpha )}\left( bx+\frac{a}{b}i+j,a^{2}z,c,\lambda \right) G_{k}^{(\alpha )}(by;c,\lambda )\right) \frac{t^{n}}{n!}. \end{aligned}$$

Our assertion follows from comparing the coefficients of $\frac{t^{n}}{n!}$ on the right hand sides of the above.□

### **Theorem 12**

*For each pair of integers a and b and*$n\ge 0$, *the following identity holds true:*

$$\begin{aligned}&\sum \limits _{k=0}^{n}\left( {\begin{array}{c}n\\ k\end{array}}\right) a^{n-k}b^{k}\sum \limits _{i=0}^{a-1}\sum \limits _{j=0}^{b-1}(-\lambda )^{i+j}{}_{H}G_{n-k}^{(\alpha )}\left( bx+\frac{ b}{a}i,b^{2}z;c,\lambda \right) G_{k}^{(\alpha )}(ay+\frac{a}{b}j;c;\lambda )\\&\quad =\sum \limits _{k=0}^{n}\left( {\begin{array}{c}n\\ k\end{array}}\right) b^{n-k}a^{k}\sum \limits _{i=0}^{b-1}\sum \limits _{j=0}^{a-1}(-\lambda )^{i+j}{}_{H}G_{n-k}^{(\alpha )}\left( ax+\frac{ a}{b}i,a^{2}z;c;\lambda \right) G_{k}^{(\alpha )}(by+\frac{b}{a}j;c;\lambda ) . \end{aligned}$$

### Proof

The proof is similar to that of Theorem 10. So we omit the proof of the theorem.□

### **Corollary 13**

*By setting*$y=0$*in Theorem*12, *we have*

$$\begin{aligned}&\sum \limits _{k=0}^{n}\left( {\begin{array}{c}n\\ k\end{array}}\right) a^{n-k}b^{k}\sum \limits _{i=0}^{a-1}\sum \limits _{j=0}^{b-1}(-\lambda )^{i+j}{}_{H}G_{n-k}^{(\alpha )}\left( bx+\frac{ b}{a}i,b^{2}z,c,\lambda \right) G_{k}^{(\alpha )}(\frac{a}{b}j,c;\lambda ) \\&\quad =\sum \limits _{k=0}^{n}\left( {\begin{array}{c}n\\ k\end{array}}\right) b^{n-k}a^{k}\sum \limits _{i=0}^{b-1}\sum \limits _{j=0}^{a-1}(-\lambda )^{i+j}{}_{H}G_{n-k}^{(\alpha )}\left( ax+\frac{ a}{b}i,a^{2}z,c,\lambda \right) G_{k}^{(\alpha )}(\frac{b}{a}j,c;\lambda ) . \end{aligned}$$

### **Theorem 13**

*Leta*, *bandcbe positive integers, by*$a\ne b$. *Then, for*$x,y\in{\mathbb{R}}$*and*$n\ge 0$, *we have*

$$\begin{aligned}&\sum \limits _{k=0}^{n}\left( {\begin{array}{c}n\\ k\end{array}}\right) b^{n-k}a^{k}G_{n-k}^{(\alpha )}(ay;c,\lambda )\sum \limits _{i=0}^{a-1}(-\lambda )^{i}{}_{H}G_{k}^{(\alpha )}\left( bx+\frac{b}{a}i,b^{2}z;c;\lambda \right) \\&\quad =\sum \limits _{k=0}^{n}\left( {\begin{array}{c}n\\ k\end{array}}\right) a^{n-k}b^{k}G_{n-k}^{(\alpha )}(by;c,\lambda )\sum \limits _{i=0}^{b-1}(-\lambda )^{i}{}_{H}G_{k}^{(\alpha )}\left( ax+\frac{a}{b}i,a^{2}z;c,\lambda \right) . \end{aligned}$$

### Proof

Let

$$g(t)=\frac{(2at)^{\alpha }(2bt)^{\alpha }(1+\lambda (-1)^{a+1}c^{abt})c^{ab(x+y)t+a^{2}b^{2}zt^{2}}}{(\lambda c^{at}+1)^{\alpha }(\lambda c^{bt}+1)^{\alpha +1}}.$$

Then we have

$$\begin{aligned} g(t) & = \left( \frac{2at}{\lambda c^{at}+1}\right) ^{\alpha }c^{abxt+a^{2}b^{2}zt^{2}}\left( \frac{1-\lambda (-c^{bt})^{a}}{\lambda c^{bt}+1}\right) \left( \frac{2bt}{\lambda c^{bt}+1}\right) ^{\alpha }c^{abyt} \\ & = \sum \limits _{k=0}^{\infty }\sum \limits _{i=0}^{a-1}(-\lambda )^{i}{}_{H}G_{k}^{(\alpha )}\left( bx+\frac{b}{a}i,b^{2}z;c,\lambda \right) \frac{a^{k}}{k!}\sum \limits _{n=0}^{\infty }G_{n}^{(\alpha )}(ay;c,\lambda )b^{n}\frac{t^{n+k}}{n!} \end{aligned}$$

from which we see that

$$\begin{aligned} I_{1}=\sum \limits _{n=0}^{\infty }\left( \sum \limits _{k=0}^{n}\left( {\begin{array}{c}n\\ k\end{array}}\right) b^{n-k}a^{k}\sum \limits _{i=0}^{a-1}(-\lambda )^{i}{}_{H}G_{k}^{(\alpha )}\left( bx+\frac{b}{a}i,b^{2}z;c,\lambda \right) G_{n-k}^{(\alpha )}(ay;c,\lambda )\right) \frac{t^{n}}{n!} \end{aligned}$$

and

$$\begin{aligned} I_{2}=\sum \limits _{n=0}^{\infty }\left( \sum \limits _{k=0}^{n}\left( {\begin{array}{c}n\\ k\end{array}}\right) a^{n-k}b^{k}\sum \limits _{i=0}^{b-1}(-\lambda )^{i}{}_{H}G_{k}^{(\alpha )}\left( ax+\frac{a}{b}i,a^{2}z,c,\lambda \right) G_{n-k}^{(\alpha )}(by;c,\lambda )\right) \frac{t^{n}}{n!}. \end{aligned}$$

Hence we complete the proof of the theorem by the equality $I_{1}=I_{2}$.□

## Conclusion

In this paper, we have introduced a new family of Apostol Hermite–Genocchi polynomials based on modified Milne-Thomson’s polynomial earlier defined by Dere and Simsek (2015). We have analysed the properties of these polynomials according to familiar properties of Apostol Hermite–Genocchi polynomials given by Gaboury and Kurt (2012) and Kurt and Kurt (2011). Also we have derived the general symmetric identities arising from different analytical means and generating functions method.
